# Temporal Patterns in Fine Particulate Matter Time Series in Beijing: A Calendar View

**DOI:** 10.1038/srep32221

**Published:** 2016-08-26

**Authors:** Jianzheng Liu, Jie Li, Weifeng Li

**Affiliations:** 1 Department of Urban Planning and Design, Faculty of Architecture, The University of Hong Kong, Hong Kong; 2 Shenzhen Institute of Research and Innovation, The University of Hong Kong, Shenzhen, China

## Abstract

Extremely high fine particulate matter (PM_2.5_) concentration has become synonymous to Beijing, the capital of China, posing critical challenges to its sustainable development and leading to major public health concerns. In order to formulate mitigation measures and policies, knowledge on PM_2.5_ variation patterns should be obtained. While previous studies are limited either because of availability of data, or because of problematic a priori assumptions that PM_2.5_ concentration follows subjective seasonal, monthly, or weekly patterns, our study aims to reveal the data on a daily basis through visualization rather than imposing subjective periodic patterns upon the data. To achieve this, we conduct two time-series cluster analyses on full-year PM_2.5_ data in Beijing in 2014, and provide an innovative calendar visualization of PM_2.5_ measurements throughout the year. Insights from the analysis on temporal variation of PM_2.5_ concentration show that there are three diurnal patterns and no weekly patterns; seasonal patterns exist but they do not follow a strict temporal division. These findings advance current understanding on temporal patterns in PM_2.5_ data and offer a different perspective which can help with policy formulation on PM_2.5_ mitigation.

Beijing, the capital of China where more than 20 million people reside, would probably never have considered it would gain the title “Capital of Smog” that was used for London 60 years ago. But now the title seems to fit Beijing appropriately. Clearly, air pollution not only undermines the reputation of Beijing as a historic world-renowned city, but more importantly it poses citizens and the government with a critical challenge for the sustainable development of urbanization that involve major public health concerns. Of all the most common detrimental air pollutants, fine particulate matter (PM_2.5_) is believed to be the most serious pollutant due to its harmful health impact on the cardiovascular, respiratory, and pulmonary functionality in humans^1^,^2^. There are increasing evidences that PM_2.5_ is associated with population mortality^3^,^4^, cardiovascular and respiratory diseases mortality^5^, and has adverse impacts on growth of new-borns^6^, and even on mental health and can cause anxiety^7^.

Actions need to be taken to mitigate PM_2.5_ problems in Beijing as well as in other cities of China. For this, we should first measure PM_2.5_ and analyse it to determine inherent variation patterns. Until now, a handle of research efforts have been made to this end^8^,^9^,^10^,^11^,^12^,^13^,^14^,^15^,^16^. While these studies report interesting results about PM_2.5_, they are defected in several ways. Some research are limited because of data availability such as a limited number of PM_2.5_ monitoring stations^8^,^11^, or that data is only available for a limited period^12^,^13^,^14^. These studies fail to provide a sufficient overview of PM_2.5_ concentration patterns across the city of Beijing and through a full year. There have been studies that analysed PM_2.5_ measurements data in a full year across Beijing that were provided by the newly launched air pollution monitoring network since later 2012^9^,^10^,^15^,^16^, but these studies, when analysing the temporal variation of PM_2.5_ concentration, used an a priori assumption that PM_2.5_ concentration follows seasonal, monthly, or weekly patterns. The reasoning in these studies is that since the PM_2.5_ concentration probably follows seasonal, monthly, or weekly patterns, the analysis framework could be based on an imposed seasonal, monthly, or weekly profile analysis. We argue that the variation of PM_2.5_ concentration may vary on different time scales other than these predefined scales, and studies using these predefined time scales are likely to provide incomplete information, and therefore miss important insights.

Our study, instead of making arbitrary assumptions on weekly, monthly, and seasonal patterns, prefers to reveal the data. Using a full year of PM_2.5_ ground-level measurements from January 2014 to December 2014 in Beijing, our study conducted two time-series clustering analyses for all the daily PM_2.5_ measurements. In this way, our study offers an innovative calendar visualization of PM_2.5_ concentration on a daily basis over the year of 2014, which yields important insights on temporal variation patterns of PM_2.5_ concentration.

The contribution of our study is two-fold. First, our study presents an innovative and straightforward calendar visualization of daily PM_2.5_ time-series in Beijing in the year of 2014. This technique provides a very useful tool to visualize and understand the data and can be applied to examine temporal patterns of other air pollutants. Second, the insights generated from the two calendar plots advance our understanding of Beijing’s PM_2.5_ concentration. Compared to previous studies on Beijing’s PM_2.5_ concentration, our study offers a different perspective and brings in insights on PM_2.5_ concentration that are more complete and convincing.

## Results and Discussion

**Figure 1:**
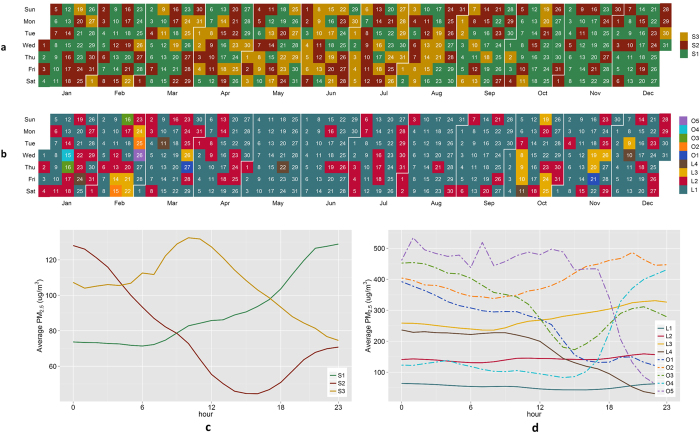
Calendar views of PM_2.5_ concentration clusters in Beijing in the year 2014. (**a**) shows PM_2.5_ time-series cluster result based on correlation distance, and the letter S denotes “shape”; (**b**) shows the cluster result based on Euclidean distance, L denotes “level” and O refers to “outlier”. (**c**) shows the averaged PM_2.5_ trend for clusters based on correlation distance, and (**d**) shows the averaged PM_2.5_ for clusters based on Euclidean distance. Note that the colours and labels are matched for each cluster for consistency, and the lines for O1, O2, O3, O4, and O5 are set to dash for clear presentation.

Figure 1 shows two calendar views of the cluster analyses using the correlation distance and Euclidean distance, and two corresponding trend curves of averaged PM_2.5_ concentrations. We obtain three clusters for the analysis based on correlation distance, and each of them has 162, 117, and 86 time-series (days). We named these as S1, S2, and S3, as shown in Fig. 1a,c. For the cluster analysis based on Euclidean distance, nine clusters are formed, each consisting of 255, 82, 15, 5, 2, 2, 2, 1, and 1 time-series. They are named as L1, L2, L3, L4, O1, O2, O3, O4, and O5 (Fig. 1b,d). Those clusters with less than three time-series, namely O1, O2, O3, O4, and O5, are considered as “outliers” that either have extremely high PM_2.5_ concentration or exhibit odd variation patterns. We will discuss these “outliers” later.

### Interpretation on calendar visualization

The calendar plot based on correlation distance (Fig. 1a) and the corresponding curve (Fig. 1c) shows the cluster result based on shape differences among the 365 PM_2.5_ time-series. The result shows that there are three distinct variation patterns for the PM_2.5_ time-series. An increasing pattern from 0 AM to 11 PM in a day is most likely to be observed from January to March and from September to December (S1 in Fig. 1a,c). For these days that show an increasing PM_2.5_ concentration pattern, the maximum PM_2.5_ concentration of the day usually occurs at night. The decreasing pattern can be observed in all months throughout the year (S2 in Fig. 1a,c) and this pattern attains its minimum value in the afternoon. The third pattern with a shape like an inverted V often take place from April to August (S3 in Fig. 1a,c) and the PM_2.5_ concentrations during these days usually peaks at noon. These results show that the diurnal patterns of PM_2.5_ vary from day to day through the year, and PM_2.5_ concentration in the daytime could be higher than at night in many days, which complement previous studies concluding that diurnal variation of PM_2.5_ change by seasons and PM_2.5_ concentration at night is higher than that in the daytime^10^,^16^.

Our findings are consistent with a previous research which identified a ‘sawtooth cycle’ of PM_2.5_ variation^17^. During a ‘sawtooth cycle’, the PM_2.5_ concentration first rises over a few days, which corresponds to the increasing pattern in our study (S1 in Fig. 1a,c), and then falls, which matches the decreasing pattern in our study (S2 in Fig. 1a,c)^17^. One possible interpretation is that the increasing and decreasing patterns (S1 and S2 in Fig. 1a,c) are largely formulated by the passage of cold front. When the cold front arrives, high-speed wind associated with the cold front blows the pollution away and thus the PM_2.5_ concentration is decreasing. But when the cold front moves on, cold air underlies the warm air as the cold air is denser and heavier, which leads to temperature inversion. The temperature inversion traps PM_2.5_ pollution near the surface and makes the PM_2.5_ concentration increasing.

Human activities such as heating and combustion, as well as weather conditions including wind, boundary layer height, etc. are closely linked to the variation of PM_2.5_ concentration^18^,^19^. As we can see in Fig. 1a,c, not all variation patterns in PM_2.5_ concentration match the daily cycle of human activities such as transportation that usually peaks in the morning and afternoon during a full day. The third pattern (S3 in Fig. 1a,c) is the closest one that possibly matches the daily cycle of human activities but this pattern usually happens from April to August. This finding suggests that the effect of human activities on variations of PM_2.5_ concentration may vary at different time periods. We speculate that from January to March and September to December, weather conditions including cold front, wind, boundary layer height, etc., may be the major factors determining variations in PM_2.5_ concentration. However, from April to August, the weather conditions (e.g., cold front) weaken and human activities thus might have stronger impact on PM_2.5_ variation.

The cluster result based on differences in PM_2.5_ concentration levels can be found in the calendar plot based on Euclidean distance (Fig. 1b) and the corresponding curve (Fig. 1d). We can see that a majority of days in the year have an averaged PM_2.5_ concentration of around 50 μg/m^3^ (L1 in Fig. 1b,d), a figure far beyond the WHO (25 μg/m^3^) and USA air quality standards (15 μg/m^3^). The calendar plot also indicates that high averaged PM_2.5_ concentration around 150 μg/m^3^ (L2 in Fig. 1b,d) are likely to occur in every month throughout the year. Also, extremely high PM_2.5_ concentration above 250 μg/m^3^ (L3, O1, O2, O3, O4, and O5 in Fig. 1b,d) can be usually observed in January, February, March, October, November, and December. This finding is consistent with previous studies concluding that PM_2.5_ concentration is generally the highest during winter and lowest during summer^15^,^16^.

### Outliers

A few “outliers” (O1, O2, O3, O4, and O5 in Fig. 1b,d) can be found in Fig. 1b. For example, two notable “outliers” O4 and O5 on January 15 and February 26, 2014, respectively, show quite drastic variations across the day. As we can see, extremely high PM_2.5_ concentrations (O5 has a maximum PM_2.5_ concentration of 534 μg/m^3^) are observed on the two days and the two incidents were reported by the Guardian^20^, Time magazine^21^, and Financial Times^22^.

One event of particular interest is the Asia-Pacific Economic Cooperation (APEC) Summit on 10 and 11 November 2014 in Beijing. It is reported that in order to maintain a blue sky in Beijing during the APEC Summit, coordinated efforts were taken by the governments of Beijing and six surrounding provinces before the summit^23^. Measures included impositions on road traffic and plant operations. The two calendar visualization plots in our study indicate that PM_2.5_ concentration was very high in mid-October before the summit. For example, on October 19, 24, and 25, the PM_2.5_ concentration was over 150 μg/m^3^. After the emission control measures were enforced, the PM_2.5_ concentration was greatly reduced on November 1. However, on November 4, a sharp increase in PM_2.5_ concentration was observed, which was around 150 μg/m^3^. Fortunately, a significant reduction occurred on November 5 and PM_2.5_ concentration returned to lower level afterwards by November 15, four days after the summit. These interpretations from the two calendar plots can also be obtained from local observations, but here we would like to note that the two calendar visualizations in our study offer a much more straightforward understanding of the whole picture of PM_2.5_ variations over time than using other tools.

### Seasonal and weekly patterns?

As we can see from the two cluster results, both shape and level variation do exhibit a rough seasonal pattern but the pattern do not follow strict seasonal divisions. As Fig. 1a shows, S1 pattern usually occurs in around winter seasons (from January to March and from September to December) and S3 patterns often happens around summer times (from April to August). Figure 1b shows a rough seasonal pattern too. Days in L3 cluster usually occur near winter (in February, March, October, November and December but not January) although days in L1 and L2 clusters can be found in any month throughout the full year which doesn’t exhibit very clear seasonal pattern. There may exist significant differences in PM_2.5_ concentration levels between different seasons^10^,^15^,^16^, however we argue that the arbitrary seasonal division of variation in PM_2.5_ concentration may result in information loss and conceal potentially important insights. The calendar visualization used in our study, however, provides an informative and straightforward way to look into variation patterns of air pollutants.

Several studies reported that there existed weekly patterns in PM_2.5_ concentration in Beijing^9^,^13^. And their findings are not consistent with each other. One study stated that the lowest concentrations occurred in Mondays while the highest concentrations appeared from Thursdays to Saturday^9^; another study concluded that PM_2.5_ concentrations on weekdays were lower than that on weekends^13^. Our findings, however, do not observe these reported weekly patterns. Figure 1b shows that among all 52 weeks in 2014, higher PM_2.5_ concentrations in weekdays than those in weekends are observed in at least 18 weeks. For example from March 24 to 30, the lowest PM_2.5_ concentrations were observed on weekends while the highest were on weekdays (Fig. 1b). Further calculations show that about half of all weeks in 2014 have higher averaged concentrations on weekdays, and the lowest PM_2.5_ concentrations are observed on Mondays of only 13 weeks and in only 30 weeks the highest concentrations appeared from Thursday to Saturday. Our results do not support the reported weekly patterns.

We did not observe any explicit and universal weekly variation pattern after visual inspection over the two calendar plots (Fig. 1a,b) and further calculations. This finding suggests that the weekly cycle of human activities may not play a key role in determining variations in PM_2.5_ concentration. Our finding complements and improves previous studies that report weekly patterns in PM_2.5_ concentration in Beijing^9^,^13^.

### Future research

As we know, PM_2.5_ pollution can be measured in terms of optical properties and chemical compositions in addition to the mass concentration^24^,^25^,^26^,^27^. With the help of the calendar visualization technique used in this study, these informative properties and other air pollutants such as NO_2_, SO_2_, and O_3_ can help provide a better understanding of the air pollution problem.

## Data and Methods

### Data

**Figure 2:**
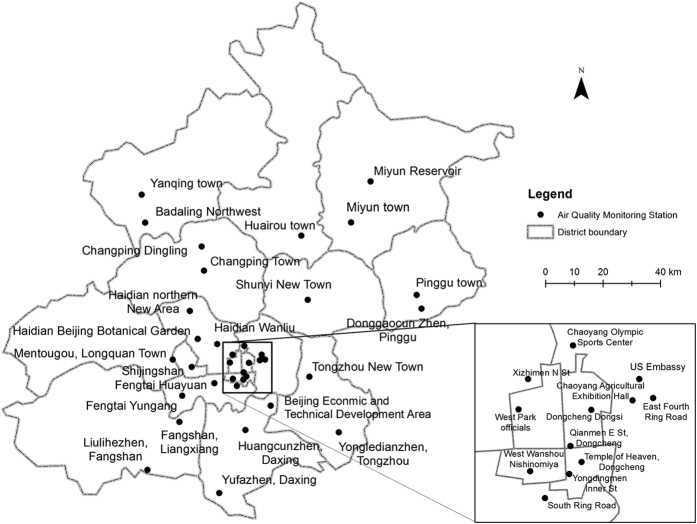
Air Quality Monitoring Stations in Beijing. The map is generated by the authors using ArcGIS 10.2.2 (www.esri.com).

The PM_2.5_ measurement data in Beijing used in this study were originally obtained from the official hourly air quality reporting platform (http://zx.bjmemc.com.cn/) run by Beijing Environment Protection Agency. This platform is part of the national air quality monitoring network initiated in late 2012. The data is rich, reporting hourly concentrations of six pollutants: particulate matter with aerodynamic diameter no greater than 2.5 microns (PM_2.5_), particulate matter with aerodynamic diameter less than 10 microns (PM_10_), and sulphur dioxide (SO_2_), nitrogen dioxide (NO_2_), ozone (O_3_), and carbon monoxide (CO) in 35 stations across Beijing (Fig. 2). However, the data is not easily accessible because the online reporting system only reports the air quality of the day and does not show historical data and is unavailable to the public. Fortunately, third parties created by civic efforts such as PM25.in, AQISTUDY.cn, and EPMAP.org have been crawling this data since late 2013.

Our study uses one-year air quality monitoring data from 1 January 2014 to 31 December 2014 from AQISTUDY.cn, EPMAP.org, and the US Embassy Beijing Air Quality Monitor (Fig. 2). We noticed that there are missing hourly measurements in all the three data sources. Therefore, we combined them to get complete PM_2.5_ measurement data covering 24 hours of all the 365 days in 2014. The US Embassy Beijing Air Quality Monitor is operated by the US Department of State. The US Department of State requires that the following disclaimer by included in any publication that uses these data: “State Air observational data are not fully verified or validated; these data are subject to change, error, and correction. The data and information are in no way official”.

A comprehensive data quality check on the raw data is conducted to reduce the impact of problematic data points, including duplicated data records, missing measurements with a placeholder, implausible zeros, etc. After the data quality check, the hourly PM_2.5_ measurement data for all 35 stations are then aggregated into one averaged PM_2.5_ concentration per hour for cluster analysis as explained below.

### Method

Since we have 24 hourly PM_2.5_ measurements for each day, it implies we have 365 time-series objects with 24 data points each to analyse. We would like to aggregate together time-series objects with similar variation patterns of PM_2.5_ concentration and separate those with dissimilar patterns into different groups. Thus, we employ time-series clustering technique to mine the data.

In general, there are two essential components in a clustering analysis: clustering algorithm and distance measure^28^. Clustering algorithm controls the procedures on how similar objects are clustered, while distance measures are used to establish the resemblance between two objects. There are several algorithm and distance measures available in the field of cluster analysis but our study employed the most straightforward and suitable clustering method and metrics. Specifically, we use average-linkage agglomerative hierarchical clustering as the clustering method because this method generates repeatable and consistent results and does not require the number of clusters to be specified as compared with K-means^29^, and it is usually able to obtain more robust cluster results than other hierarchical clustering methods^30^.

Distance measures were selected based on two basic features of the PM_2.5_ time-series data: level and shape. Level refers to the quantity of PM_2.5_ concentration, and the Euclidean distance is used to identify the level difference between PM_2.5_ time-series. Shape refers to trends in PM_2.5_ concentration variation with respect to time, and we use Pearson’s correlation-based distance to capture the shape difference between PM_2.5_ time-series. We derived a generalized correlation-based dissimilarity function from this study^31^ by making the coefficient *α* and power *β* adjustable (equation (1)).


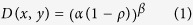


where the correlation coefficient
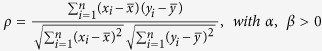


This dissimilarity function satisfies all the requirements for dissimilarity measure: the non-negativity, symmetry, and identity^32^,^33^. When both *α* and *β* are set to 1, this dissimilarity function becomes the classic Pearson’s correlation coefficient distance that has been used in several studies^34^. In our study, however, we deliberately set *α* and *β* to 0.5 and 0.25, respectively, in order to attain a desirable robust cluster result.

We employ the cophenetic correlation coefficient to examine the validity and robustness of the cluster analysis. Cophenetic correlation coefficient is a measure of how faithfully the hierarchical cluster results represent the dissimilarity among observations^35^. It is defined as the linear correlation coefficient between the original pairwise dissimilarities and the cophenetic dissimilarities obtained from the dendrogram. The value of this coefficient varies between 0 and 1. A higher cophenetic correlation coefficient indicates a better cluster solution, and a value of 0.8 or higher is usually regarded as a successful cluster application^36^.

It turns out that the cophenetic correlation coefficients for Euclidean-distance-based and correlation-distance-based cluster analyses are 0.86 and 0.81, respectively, suggesting that both cluster results are robust and valid.

We used Python version 2.7.5 to process and analyse the data, and R version 3.2.2 to draw the calendar plots.

## Additional Information

**How to cite this article**: Liu, J. *et al*. Temporal Patterns in Fine Particulate Matter Time Series in Beijing: A Calendar View. *Sci. Rep.* **6**, 32221; doi: 10.1038/srep32221 (2016).

## References

[b1] Yuan Y., Liu S., Castro R. & Pan X. PM2.5 monitoring and mitigation in the cities of China. Environmental science & technology 46, 3627–3628, 10.1021/es300984j (2012). 22448594

[b2] Han L., Zhou W. & Li W. Increasing impact of urban fine particles (PM2.5) on areas surrounding Chinese cities. Scientific reports 5, 12467, 10.1038/srep12467 (2015). 26219273 PMC4518225

[b3] Wong C. M. . Satellite-Based Estimates of Long-Term Exposure to Fine Particles and Association with Mortality in Elderly Hong Kong Residents. Environ Health Perspect 123, 1167–1172, 10.1289/ehp.1408264 (2015). 25910279 PMC4629733

[b4] Samet J. M., Dominici F., Curriero F. C., Coursac I. & Zeger S. L. Fine particulate air pollution and mortality in 20 US cities, 1987–1994. New England journal of medicine 343, 1742–1749 (2000). 11114312 10.1056/NEJM200012143432401

[b5] Dominici F. . Fine particulate air pollution and hospital admission for cardiovascular and respiratory diseases. JAMA: the journal of the American Medical Association 295, 1127–1134 (2006). 16522832 10.1001/jama.295.10.1127 PMC3543154

[b6] Jedrychowski W. . Gender differences in fetal growth of newborns exposed prenatally to airborne fine particulate matter. Environ. Res. 109, 447–456 (2009). 19261271 10.1016/j.envres.2009.01.009 PMC3786262

[b7] Power M. C. . The relation between past exposure to fine particulate air pollution and prevalent anxiety: observational cohort study. BMJ 350, h1111, 10.1136/bmj.h1111 (2015). 25810495 PMC4373600

[b8] Streets D. G. . Air quality during the 2008 Beijing Olympic Games. Atmospheric Environment 41, 480–492, 10.1016/j.atmosenv.2006.08.046 (2007).

[b9] Chen W., Tang H. & Zhao H. Diurnal, weekly and monthly spatial variations of air pollutants and air quality of Beijing. Atmospheric Environment 119, 21–34 (2015).

[b10] Huang F. . PM2.5 Spatiotemporal Variations and the Relationship with Meteorological Factors during 2013–2014 in Beijing, China. PLoS One 10, e0141642 (2015). 26528542 10.1371/journal.pone.0141642 PMC4631325

[b11] Zheng S., Pozzer A., Cao C. & Lelieveld J. Long-term (2001–2012) concentrations of fine particulate matter (PM2.5) and the impact on human health in Beijing, China. Atmospheric Chemistry and Physics 15, 5715–5725 (2015).

[b12] Chai F. . Spatial and temporal variation of particulate matter and gaseous pollutants in 26 cities in China. Journal of Environmental Sciences 26, 75–82, 10.1016/S1001-0742(13)60383-6 (2014). 24649693

[b13] Hu J., Wang Y., Ying Q. & Zhang H. Spatial and temporal variability of PM2.5 and PM10 over the North China plain and the Yangtze River delta, China. Atmospheric Environment 95, 598–609, 10.1016/j.atmosenv.2014.07.019 (2014).

[b14] Rohde R. A. & Muller R. A. Air Pollution in China: Mapping of Concentrations and Sources. PLoS One 10, e0135749, 10.1371/journal.pone.0135749 (2015). 26291610 PMC4546277

[b15] Wang Y., Ying Q., Hu J. & Zhang H. Spatial and temporal variations of six criteria air pollutants in 31 provincial capital cities in China during 2013–2014. Environ. Int. 73, 413–422, 10.1016/j.envint.2014.08.016 (2014). 25244704

[b16] Zhang Y.-L. & Cao F. Fine particulate matter (PM2.5) in China at a city level. Scientific Reports 5, 14884, 10.1038/srep14884 (2015). 26469995 PMC4606739

[b17] Jia Y., Rahn K. A., He K., Wen T. & Wang Y. A novel technique for quantifying the regional component of urban aerosol solely from its sawtooth cycles. Journal of Geophysical Research: Atmospheres (1984–2012) 113, 10.1029/2008JD010389 (2008).

[b18] Wang J., Hu Z., Chen Y., Chen Z. & Xu S. Contamination characteristics and possible sources of PM10 and PM2. 5 in different functional areas of Shanghai, China. Atmospheric Environment 68, 221–229 (2013).

[b19] Zhao X. . Seasonal and diurnal variations of ambient PM2.5 concentration in urban and rural environments in Beijing. Atmospheric Environment 43, 2893–2900, 10.1016/j.atmosenv.2009.03.009 (2009).

[b20] Gardner D. *Beijing’s smog is increasingly toxic for China’s politicians*. (2014) Available at: http://www.theguardian.com/environment/2015/jan/20/beijings-smog-increasingly-toxic-chinas-politicians (Accessed: 9 December 2015).

[b21] Beech H. *China’s Smog Is So Bad They’re Now Calling It a ‘Nuclear Winter’*. (2014) Available at: http://time.com/9802/beijing-air-pollution-nuclear-winter/. (Accessed: 9 December 2015).

[b22] Hornby L. *China pollution: Trouble in the air*. (2014) Available at: http://on.ft.com/1bMwV4d (Accessed: 9 December 2015).

[b23] Zheng S. R. *Beijing Smog: The Day After ‘APEC Blue’*. (2014) Available at: http://thediplomat.com/2014/11/beijing-smog-the-day-after-apec-blue/ (Accessed: December 9 2015).

[b24] Wang X., Huang J., Zhang R., Chen B. & Bi J. Surface measurements of aerosol properties over northwest China during ARM China 2008 deployment. Journal of Geophysical Research: Atmospheres 115, 10.1029/2009JD013467 (2010).

[b25] Zhang R. . Chemical characterization and source apportionment of PM2.5 in Beijing: seasonal perspective. Atmos. Chem. Phys. 13, 7053–7074, 10.5194/acp-13-7053-2013 (2013).

[b26] Bi J., Huang J., Hu Z., Holben B. & Guo Z. Investigating the aerosol optical and radiative characteristics of heavy haze episodes in Beijing during January of 2013. Journal of Geophysical Research: Atmospheres 119, 9884–9900 (2014).

[b27] Huang R.-J. . High secondary aerosol contribution to particulate pollution during haze events in China. Nature 514, 218–222, 10.1038/nature13774 (2014). 25231863

[b28] Wang X., Smith K. & Hyndman R. Characteristic-based clustering for time series data. Data mining and knowledge Discovery 13, 335–364 (2006).

[b29] Aghabozorgi S., Seyed Shirkhorshidi A. & Ying Wah T. Time-series clustering – A decade review. Information Systems 53, 16–38, 10.1016/j.is.2015.04.007 (2015).

[b30] Rodrigues F. M. & Diniz-Filho J. A. F. Hierarchical structure of genetic distances: effects of matrix size, spatial distribution and correlation structure among gene frequencies. Genetics and Molecular Biology 21, 233–240 (1998).

[b31] Golay X. . A new correlation‐based fuzzy logic clustering algorithm for FMRI. Magnetic Resonance in Medicine 40, 249–260 (1998). 9702707 10.1002/mrm.1910400211

[b32] Veltkamp R. C. Shape matching: Similarity measures and algorithms. Paper presented at *SMI 2001 International Conference on Shape Modeling and Applications*, Genova. IEEE. doi: 10.1109/SMA.2001.923389 (2001, May 7).

[b33] Wang L., Yang C. & Feng J. On learning with dissimilarity functions. Paper presented at *Proceedings of the 24th international conference on Machine learning*, Corvallis, Oregon, USA. New York, NY, USA: ACM. doi: 10.1145/1273496.1273621 (2007, June 20).

[b34] Jaskowiak P. A., Campello R. J. & Costa I. G. On the selection of appropriate distances for gene expression data clustering. BMC bioinformatics 15, 10.1186/1471-2105-15-S2-S2 (2014). PMC4072854 24564555

[b35] Sokal R. R. & Rohlf F. J. The comparison of dendrograms by objective methods. Taxon 11, 33–40 (1962).

[b36] Romesburg C. Cluster analysis for researchers (Lulu Press, 2004).

